# Cardioprotective effects of fatty acid amide hydrolase inhibitor URB694, in a rodent model of trait anxiety

**DOI:** 10.1038/srep18218

**Published:** 2015-12-14

**Authors:** Luca Carnevali, Federica Vacondio, Stefano Rossi, Emilio Macchi, Gilberto Spadoni, Annalida Bedini, Inga D. Neumann, Silvia Rivara, Marco Mor, Andrea Sgoifo

**Affiliations:** 1 Department of Neuroscience, University of Parma, Italy; 2 Department of Pharmacy, University of Parma, Italy; 3 Department of Life Sciences, University of Parma, Italy; 4 Department of Biomolecular Sciences, University of Urbino “Carlo Bo”, Italy; 5 Department of Behavioural and Molecular Neurobiology, University of Regensburg, Germany

## Abstract

In humans, chronic anxiety represents an independent risk factor for cardiac arrhythmias and sudden death. Here we evaluate in male Wistar rats bred for high (HAB) and low (LAB) anxiety-related behavior, as well as non-selected (NAB) animals, the relationship between trait anxiety and cardiac electrical instability and investigate whether pharmacological augmentation of endocannabinoid anandamide-mediated signaling exerts anxiolytic-like and cardioprotective effects. HAB rats displayed (i) a higher incidence of ventricular tachyarrhythmias induced by isoproterenol, and (ii) a larger spatial dispersion of ventricular refractoriness assessed by means of an epicardial mapping protocol. In HAB rats, acute pharmacological inhibition of the anandamide-degrading enzyme, fatty acid amide hydrolase (FAAH), with URB694 (0.3 mg/kg), (i) decreased anxiety-like behavior in the elevated plus maze, (ii) increased anandamide levels in the heart, (iii) reduced isoproterenol-induced occurrence of ventricular tachyarrhythmias, and (iv) corrected alterations of ventricular refractoriness. The anti-arrhythmic effect of URB694 was prevented by pharmacological blockade of the cannabinoid type 1 (CB_1_), but not of the CB_2_, receptor. These findings suggest that URB694 exerts anxiolytic-like and cardioprotective effects in HAB rats, the latter via anandamide-mediated activation of CB_1_ receptors. Thus, pharmacological inhibition of FAAH might be a viable pharmacological strategy for the treatment of anxiety-related cardiac dysfunction.

Chronic (trait) anxiety can be viewed as a dispositional tendency to experience an anxious state more frequently, at higher intensities and/or in inappropriate situations^1^. A consistent body of evidence suggests that chronic anxiety may play a role in both the incidence and progression of cardiovascular disease^2^,^3^,^4^. Alterations in the autonomic neural control of cardiovascular function represent a putative pathophysiological mechanism underlying this association. For example, enduring changes in the sympathovagal balance toward sympathetic hyperactivity and/or parasympathetic hypoactivity have been reported in anxious individuals^5^,^6^. These features of cardiac autonomic neural outflow are thought to bring about disturbances of myocardial repolarization, thereby lowering the threshold for arrhythmias and sudden cardiac death^5^,^7^,^8^.

Animal research has just started investigating the complex interplay between anxiety states, autonomic neural changes and electrical stability of the heart. For example, substantial differences in the regulation of cardiac autonomic function have recently been reported in two Wistar rat lines selectively bred for either high (HAB) or low (LAB) anxiety-related behavior^9^. In this study, HAB rats displayed a relatively low vagal component of heart rate variability (HRV) during resting conditions and a larger susceptibility to pharmacologically-induced ventricular tachyarrhythmias^9^. Therefore, HAB and LAB rats are a useful rodent model for investigating the cardiac electrical substrates of the increased vulnerability to arrhythmias that characterizes anxiety.

Given the increased likelihood of cardiovascular alterations in high anxious individuals, it is not only important to understand the mechanistic bases of this association, but also to develop therapeutic treatments for anxiety that could desirably improve cardiovascular function. Indeed, conventional anti-anxiety medications, such as benzodiazepines, do not seem to provide direct benefits on cardiovascular health^10^. Recent investigations have started to draw attention to the role of the endocannabinoid (ECB) system in the pathophysiology of affective disturbances such as anxiety and depression^11^,^12^. The endogenous cannabinoid ligand anandamide (AEA) activates the two major cannabinoid receptors, type 1 (CB_1_) and type 2 (CB_2_). Following rapid on-demand biosynthesis, AEA is inactivated by cellular uptake followed by intracellular hydrolysis by fatty acid amide hydrolase (FAAH), which also cleaves the noncannabinoid fatty acid ethanolamides oleoylethanolamide (OEA) and palmitoylethanolamide (PEA)^13^. Converging preclinical studies indicate that pharmacological inhibition of FAAH augments brain AEA levels and elicits anxiolytic-like effects in a CB_1_ receptor-dependent manner^14^,^15^,^16^,^17^,^18^, providing support for the potential utility of FAAH inhibitors in the treatment of anxiety disorders (for reviews see:^19^,^20^). Available data suggest that the ECB system also plays a role in the regulation of cardiac function and might be a promising therapeutic target for a variety of cardiac dysfunction conditions (for reviews see:^21^,^22^). CB_1_ and CB_2_ receptors are expressed in cardiac myocytes^23^,^24^. Preliminary preclinical evidence indicates that activation of the ECB pathway with exogenous AEA protects the heart from arrhythmias induced by adrenaline administration^25^ or ischemia-reperfusion procedure^26^ in rats. Intriguingly, chronic administration of URB694^27^, a second generation FAAH inhibitor with improved metabolic stability and selectivity^28^,^29^, has recently been shown to prevent the adverse behavioral and cardiac effects of repeated social stress exposure in rats^30^. Taken together, these findings prompt further investigation aimed at determining whether inhibition of FAAH activity may represent a viable pharmacological strategy for the treatment of the comorbidity of cardiovascular disease with anxiety and mood disorders.

Given the above reported considerations, in the present study we used the HAB/LAB rat model in order to: (i) evaluate whether high levels of trait anxiety are associated with pro-arrhythmic electrical remodeling of the ventricular myocardium and increased vulnerability to pharmacologically-induced arrhythmias, (ii) assess whether the FAAH inhibitor URB694 exerts anxiolytic-like effects and improves cardiac electrical stability, and (iii) investigate whether the cardioprotective effects of URB694 are mediated by facilitation of AEA signaling at CB_1_ and/or CB_2_ receptors.

## Methods

### Animals and ethics statement

Experimental procedures were carried out on 5-month-old male Wistar rats (350–450 g) selectively bred for high (HAB) and low (LAB) anxiety-related behavior in the elevated plus-maze test^31^ or non-selected rats (NAB). The animals were obtained from the animal facilities of the University of Regensburg (Germany), where they were tested at the age of 9 weeks on the elevated plus maze to confirm the selection criteria^31^. At their arrival in our laboratory, they were kept in rooms with controlled temperature (22 ± 2 °C) and a reversed light-dark cycle (light on from 19:00 to 7:00 h), with free access to food and water. Experiments were conducted with the approval of the Veterinarian Animal Care and Use Committee of Parma University, and carried out in accordance with the European Community Council Directives of 22 September 2010 (2010/63/UE).

### Drugs

URB694 (URB) belongs to the class of carbamate FAAH inhibitors that irreversibly carbamoylate the nucleophile catalytic serine in FAAH active site^27^. URB was prepared using an improved method that led to higher yield and easier purification compared to those originally reported^27^, following a reaction scheme that had been set up for a similar compound^32^. Physicochemical properties of URB were identical to those previously described^27^. Rimonabant (RIM, selective antagonist of CB_1_ receptors), AM630 (selective antagonist of CB_2_ receptors), and isoproterenol hydrochloride (ISOP, agonist of cardiac β-adrenoreceptors) were purchased from Sigma Aldrich (Italy). URB, RIM and AM630 were dissolved in a vehicle (VEH) of PEG-400, Tween-80, and saline solution (0.9% NaCl) (5:5:90,vol/vol/vol), while ISOP was dissolved in saline solution (0.9% NaCl). All drugs were administered intraperitoneally to rats in a volume of 0.5 ml/kg. The doses (see below) and time course of drug administration were chosen based on the available literature data^9^,^15^,^33^.

### Experiment 1

Experiment 1 was conducted in order to (i) confirm expected differences in anxiety-related behavior of HAB, NAB and LAB rats (n = 9 per group) in the elevated plus-maze test, and (ii) evaluate the anxiolytic-like effects of URB. The elevated plus-maze consisted of 4 elevated arms (100 cm above the floor, 50 cm long and 10 cm wide) arranged in a cross-like position, with two opposite arms being enclosed (by means of 40 cm high walls), and two being open, including at their intersection a central square platform (10 × 10 cm) which gave access to the four arms. Initially, rats were injected with either VEH or URB (0.3 mg/kg). Thirty min later, each rat was placed on the central platform of the plus-maze facing one closed arm and behaved freely for 5 min. The test was performed twice in the same animals 3 days apart, with the order of treatments that was balanced over experimental days. The behavior during the test was recorded using a video camera positioned above the maze. The percentage of time spent on the open arms was used as a measure of anxiety, whereas closed arm entries were considered indicators of overall locomotor activity^34^.

### Surgery

Radiotelemetric transmitters (Data Sciences International, St. Paul, MN; model TA11CTA-F40) were implanted under anhestesia (tiletamine hydrochloride + zolazepam hydrochloride, Zoletil, 20 mg/kg, s.c.) for ECG recordings in animals used for Experiment 2 and 3, using procedures described previously^35^. Following the surgical procedures, rats were individually housed, injected for 2 days with gentamicin sulfate (Aagent, Fatro, 0.2 ml/kg, s.c.) and allowed 10 days of recovery before the start of experimental recordings.

### Experiment 2

Experiment 2 was conducted in HAB, NAB and LAB rats (n = 9 per group) in order to (i) assess the vulnerability to ISOP-induced arrhythmias in animals with different levels of anxiety-related behavior, (ii) investigate electrical properties of the myocardium relevant to arrhythmogenesis, and (iii) evaluate potential cardioprotective effects of URB694.

#### Pharmacological challenge with isoproterenol

All animals were exposed to two experimental sessions conducted on different days, at least 72 h apart: (1) VEH + ISOP injections, (2) URB + ISOP injections. The sequence of injections was counterbalanced for each rat group. On each experimental day, animals first received the injection of either VEH or URB (0.3 mg/kg). Thirty min later, all animals were injected with ISOP (0.02 mg/kg).

#### Epicardial mapping protocol

At least 72 h after the last session, rats were anesthetized with medetomidine (0.4 mg/kg, i.p.) and ketamine (50 mg/kg, i.p.). Under artificial ventilation (Rodent Ventilator 7025, Ugo Basile, Comerio, Italy) the heart was exposed through a longitudinal sternotomy. An epicardial electrode array^36^ was used to record 64 unipolar epicardial electrograms from the anterior ventricular surface during normal sinus rhythm and ventricular pacing. The epicardial mapping protocol was performed 15 min after the injection of VEH and repeated again 15 min after the injection of URB (0.3 mg/kg). The following electrophysiological properties were measured:

i) *Excitability*: The heart was paced at 10 selected electrodes of the array by electrical stimuli of 1 ms duration at a frequency slightly higher than normal sinus rhythm. The intensity of the stimuli was progressively decremented until capture was lost. Thus, at each electrode position, we determined the diastolic threshold current strength (TCS), which was defined as the lowest current strength that was able to elicit a propagated response.

ii) *Refractoriness*: Ten baseline stimuli (S1), 1 ms width and twice TCS, were delivered at 5 selected electrodes of the array at a frequency slightly higher than normal sinus rhythm^37^. The S1 pacing sequence was followed by a premature stimulus (S2, four-fold S1 strength) whose delay from previous S1 was first progressively decremented by 10 ms steps until capture was lost and then progressively incremented by 2 ms steps till capture was resumed. Thus, at each electrode position, we determined the effective refractory period (ERP), which was defined as the shortest S1-S2 time interval at which excitation from S2 failed.

#### Measurements at sacrifice

Upon completion of the epicardial protocol, the hearts were removed and stored at −80 °C until analysis of FAAH activity and fatty acid ethanolamide AEA, OEA, PEA levels. Additional HAB, NAB and LAB rats (n = 8) were anesthetized with medetomidine (0.4 mg/kg, i.p.) and ketamine (50 mg/kg, i.p.). The hearts were exposed through a longitudinal sternotomy and removed for analysis of FAAH activity and AEA, OEA and PEA levels after an injection of VEH.

### Experiment 3

Experiment 3 was aimed at determining whether the anti-arrhythmic effect of URB in HAB rats was mediated by CB_1_ and/or CB_2_ receptors.

#### Pharmacological challenge with isoproterenol

HAB rats (n = 8) were exposed to four experimental sessions conducted on different days, at least 72 h apart: (1) VEH + VEH + ISOP injections, (2) VEH + URB + ISOP injections, (3) RIM + URB + ISOP injections, and (4) AM630 + URB + ISOP injections. The sequence of the four pharmacological sessions was counterbalanced in a randomized manner. URB was administered at a dose of 0.3 mg/kg, RIM and AM630 at a dose of 2.0 mg/kg and ISOP at a dose of 0.02 mg/kg. Each i.p. injection was separated from the preceding and/or the following ones by a 30-min time interval.

### Data collection and analysis

#### ECG data

During the pharmacological challenge sessions, continuous ECG recordings were performed for 30 min in baseline conditions and following each injection. HR (reported in beats per minute; bpm) and time- and frequency-domain parameters of HRV were quantified using ChartPro 5.0 software (ADInstruments, Sydney, Australia). In the time-domain, we calculated the root mean square of successive RR interval differences (RMSSD, ms), which reflects the vagal input to the heart^38^. In the frequency-domain (fast-Fourier transformation), we measured (i) the power of the low (LF; 0.2–0.75 Hz) (ms^2^) and the high (HF; 0.75–2.5 Hz) (ms^2^) frequency bands, the latter reflecting respiratory-related vagal influences^38^,^39^, and (ii) the LF to HF ratio, which is taken as a synthetic measure of sympathovagal balance^38^,^40^. In addition, the occurrence of ventricular ectopic beats was determined and quantified off-line based on the Lambeth Conventions for the study of experimental arrhythmias^41^.

#### Epicardial mapping data

i) *Excitability*: For each animal, we calculated (i) the mean TCS value as an index of overall ventricular excitability, and (ii) the standard deviation (SD) of the mean TCS value as a measure of the spatial dispersion of ventricular excitability.

ii) *Refractoriness*: For each animal, we calculated (i) the mean ERP value as an index of overall ventricular refractoriness, and (i) the SD of the mean ERP value as a measure of the spatial dispersion of ventricular refractoriness^42^.

#### Measurements at sacrifice

FAAH activity (reported in counts per minute, cpm) was quantified from atrial and ventricular homogenates at 37 °C for 30 min in 0.5 mL Tris buffer (50 mM, pH 7.5) containing fatty acid-free bovine serum albumin (BSA) (0.05%, w/v), 50 μg of protein from brain homogenates, 10 μM anandamide and [^3^H]-anandamide (10000 disintegrations per minute), following previously described procedures^28^,^30^. Fatty acid ethanolamides AEA, OEA and PEA were extracted from atrial and ventricular homogenates by organic solvent (ice-cold acetonitrile) addition and quantified by HPLC/MS/MS^30^. The HPLC/MS/MS analytical standards AEA, OEA, PEA and the deuterated internal standards, AEA-d_4_, PEA-d_4_, OEA-d_4_ were purchased from Cayman Chemicals (Ann Arbor, Michigan) as stock solutions in ethanol.

#### Statistical analyses

All statistical analyses were performed using the software package SPSS (version 21). Statistical significance was set at p < 0.05.

*Experiment 1*: Behavioral data were analyzed with 3 (HAB, NAB, or LAB group) x 2 (URB694 or VEH treatment) factorial design ANOVAs. Follow-up analyses were conducted using Student’s “t” tests, with a Bonferroni correction for multiple comparisons for each outcome variable separately.

*Experiment 2*: Two-way ANOVAs for repeated measures were applied to: (i) ECG data, with “time” as within-subject factor (3 levels: baseline, VEH/URB injection, ISOP injection) and “group” (3 levels: HAB, NAB and LAB) or “treatment” (2 levels: VEH and URB) as between-subject factors, (ii) epicardial mapping data and measurements at sacrifice with “treatment” as within-subject factor (2 levels: VEH and URB) and “group” as between-subject factor (3 levels: HAB, NAB and LAB). Follow-up analyses were conducted using Student’s “t” tests, with a Bonferroni correction for multiple comparisons for each outcome variable separately.

*Experiment 3*: Two-way ANOVAs for repeated measures were applied to ECG data, with “time” as within-subject factor (4 levels: baseline, post-injection1, post-injection2, and post-ISOP injection) and “group” as between-subject factor (4 levels). At each time point (baseline, post-injection1, post-injection2, and post-ISOP injection), ECG data were further analyzed by means of one-way ANOVA (4 groups) followed by Student’s “t” tests with a Bonferroni correction for multiple comparisons for each outcome variable separately. In order to minimize the number of interventions on the same animal, we used the least possible number of control conditions. In the figures and in the table of experiment 3, only comparisons of interest have been reported (i.e., VEH + VEH + ISOP vs. VEH + URB + ISOP group, and VEH + URB + ISOP vs. RIM + URB + ISOP vs. AM630 + URB + ISOP).

## Results

**Figure 1:**
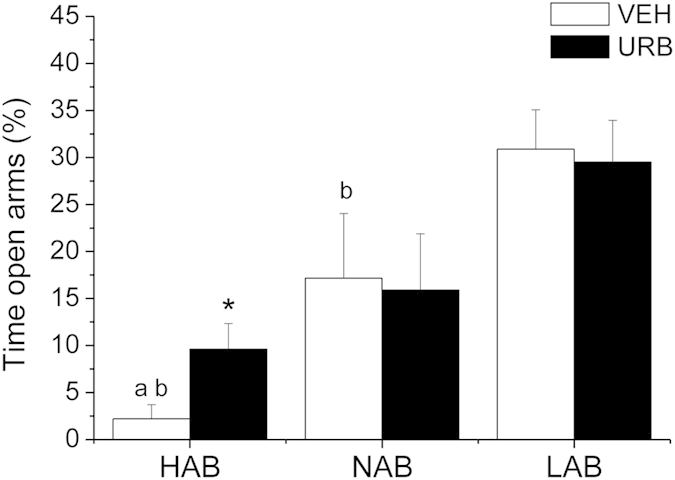
Percentage of time spent by HAB, NAB and LAB rats (n = 9 per group) on the open arms of the elevated plus maze after inhibition of FAAH activity with URB694 (URB, 0.3 mg/kg) or vehicle (VEH) administration. Data are reported as means ± SEM. ^a^vs. NAB value, ^b^vs. LAB value, *vs. respective VEH value.

**Experiment 1**. *Anxiety-related behavior*. HAB rats spent less time on the open arms of the plus-maze compared to NAB (t = −2.3, p < 0.05) and LAB (t = −6.4, p < 0.01) rats (Fig. 1). URB administration significantly increased the time spent by HAB rats on the open arms (t = 2.4, p < 0.05) compared to VEH condition (Fig. 1), without affecting overall locomotor activity (n° entries in the closed arms: VEH = 4.2 ± 0.5 vs. URB = 4.3 ± 0.7). URB treatment had no effects on NABs’ and LABs’ behavioral performance in the elevated plus maze test (Fig. 1).

**Figure 2:**
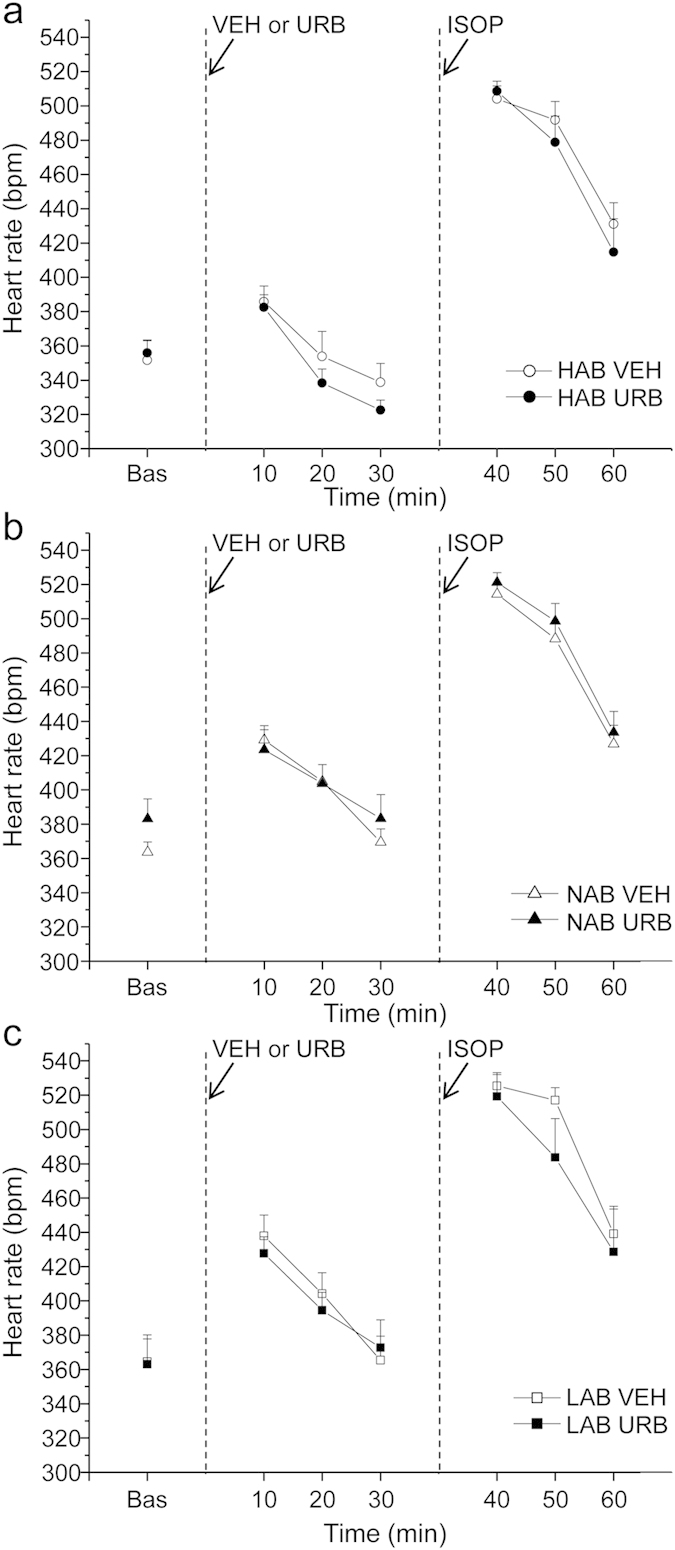
Time course of heart rate changes in HAB (panel a), NAB (panel b) and LAB (panel c) rats (n = 9 per group) in response to (i) inhibition of FAAH activity with URB694 (URB, 0.3 mg/kg) or vehicle (VEH) administration, followed by (ii) β-adrenergic stimulation with isoproterenol (ISOP, 0.02 mg/kg). Data are reported as means ± SEM of a 30-min time period for baseline conditions (bas) and three 10-min time periods after each injection.

**Experiment 2**. *Pharmacological challenge with isoproterenol*. HR and HRV changes during pharmacological challenge with ISOP in HAB, NAB and LAB rats are summarized in Table 1 and depicted in Fig. 2. Two-way ANOVA yielded a significant effect of “group” for HR values (F = 4.3, p < 0.05). Follow-up analyses revealed that baseline HR was similar in three groups (Table 1). After VEH injection, HR was significantly lower in HAB rats compared to NAB (t = −3.6, p < 0.05) and LAB (t = −2.8, p < 0.05) rats (Table 1). No differences were observed in HR response to ISOP administration between the three groups (Table 1). In addition, no significant group differences were observed in HRV parameters both in baseline conditions and following VEH and ISOP injections (Table 1). URB administration did not modify HR and HRV parameters compared to VEH administration, and did not influence HR and HRV responses to ISOP administration in any of the three groups (Fig. 2 and Table 1).

**Figure 3:**
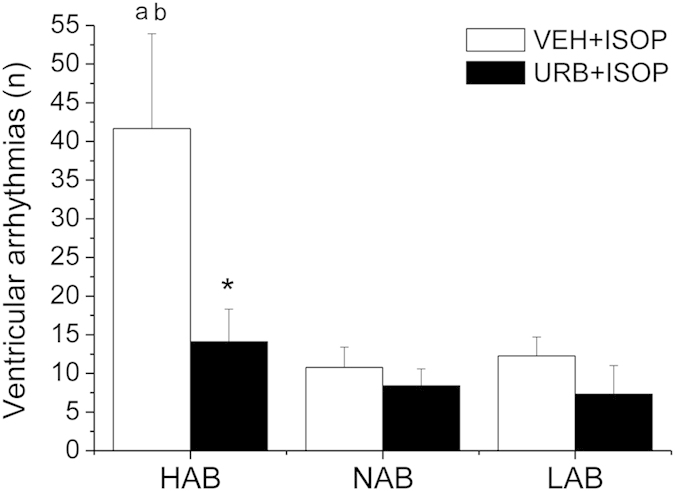
Incidence of ventricular arrhythmias after β-adrenergic stimulation with isoproterenol (ISOP, 0.02 mg/kg) in HAB, NAB and LAB rats (n = 9 per group), pretreated with either vehicle (VEH) or URB694 (URB, FAAH activity inhibitor, 0.3 mg/kg). Data are reported as means ± SEM of a 30-min time period. Significant differences (Bonferroni test, p < 0.05): ^a^vs. NAB value, ^b^vs. LAB value, *vs. respective VEH value.

The occurrence of ventricular arrhythmias after ISOP administration is represented in Fig. 3. Ectopic beats induced by ISOP administration occurred mostly as isolated ventricular events and sporadically as ventricular couplets or triplets. Two-way ANOVA yielded a significant effect of (i) “group” (F = 6.1, p < 0.01), and (ii) “treatment” (F = 6.2, p < 0.05). HAB rats showed a larger incidence of ISOP-induced ventricular arrhythmias compared to NAB (t = 2.4, p < 0.05) and LAB (t = 2.3, p < 0.05) animals (Fig. 3). Pretreatment with URB significantly reduced ISOP-induced arrhythmia occurrence in HAB rats compared to VEH pretreatment condition (t = 2.2, p < 0.05), whereas it had no significant effects in NAB and LAB rats (Fig. 3).

**Figure 4:**
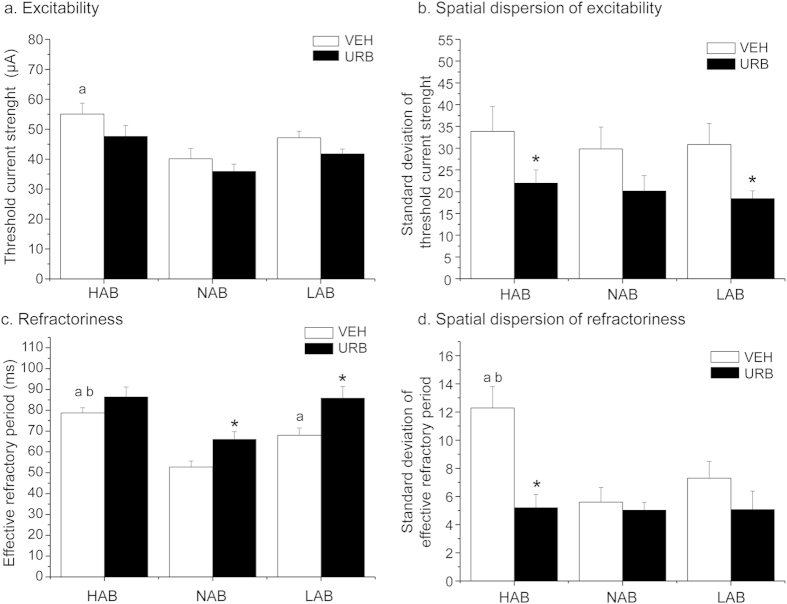
Parameters of ventricular excitability (panels a, b) and refractoriness (panels c, d) in HAB, NAB and LAB rats (n = 9 per group) after inhibition of FAAH activity with URB694 (URB, 0.3 mg/kg) or vehicle (VEH) administration. Data are reported as means ± SEM. Significant differences (Bonferroni test, p < 0.05): ^a^vs. NAB value, ^b^vs. LAB value, *vs. respective VEH value.

*Epicardial mapping protocol*. Epicardial mapping parameters in HAB, LAB and NAB rats are depicted in Fig. 4.

i) *Excitability*: Two-way ANOVA yielded (i) a significant effect of “group” for mean TCS values (F = 7.2, p < 0.01), and (ii) a significant effect of “treatment” (F = 8.9, p < 0.01) for the SD of the mean TCS. Follow-up analyses revealed that average TCS was significantly higher in HABs compared to NAB rats (t = 2.9, p < 0.05), whereas no significant differences were observed between (i) HAB and LAB rats, and (ii) NAB and LAB rats (Fig. 4a). URB administration did not significantly modify average TCS in any of the three groups (Fig. 4a). In addition, the SD of the mean TCS was similar between the three groups (Fig. 4b). URB administration significantly reduced the SD of the mean TCS in HAB (t = 2.5, p < 0.05) and LAB (t = 3.4, p < 0.01) rats compared to VEH condition (t = 4.5, p < 0.01) (Fig. 4b).

ii) *Refractoriness*: Two-way ANOVA yielded (i) significant effects of “group” (F = 11.3, p < 0.01) and “treatment” (F = 11.3, p < 0.01) for mean ERP values, and (ii) a significant effect of treatment (F = 23.6, p < 0.01) and a “group x treatment” interaction (F = 8.2, p < 0.01) for the SD of the mean ERP. Follow-up analyses revealed that average ERP was significantly longer (i) in HABs compared to NAB (t = 6.7, p < 0.01) and LAB (t = 2.5, p < 0.05) rats, and (ii) in LABs compared to NAB rats (t = 3.4, p < 0.01) (Fig. 4c). URB administration significantly increased average ERP in NAB (t = 6.2, p < 0.01) and LAB (t = 4.5, p < 0.01) rats compared to VEH values (Fig. 4c). In addition, the SD of the mean ERP was significantly larger in HABs compared to NAB (t = 3.4, p < 0.01) and LAB (t = 3.4, p < 0.01) rats, whereas no differences were observed between NAB and LAB animals (Fig. 4d). URB administration significantly reduced the SD of the mean ERP in HAB rats compared to VEH condition (t = 4.5, p < 0.01) (Fig. 4d).

*FAAH activity and AEA, OEA and PEA levels in heart homogenates*. After VEH administration, there was a significant effect of “group” in atrial and ventricular levels of FAAH activity (F_atrial_ = 8.4, p < 0.01; F_ventricular_ = 41.4, p < 0.01). URB administration considerably reduced FAAH activity levels in heart homogenates (F_atrial_ = 112.1, p < 0.01; F_ventricular_ = 265.9, p < 0.01), with no group differences (Table 2). AEA, OEA and PEA levels were significantly higher after URB treatment compared to VEH administration both in atrial (F_AEA_ = 20.1, p < 0.01; F_OEA_ = 39.8, p < 0.01; F_PEA_ = 7.7, p < 0.01) and ventricular (F_AEA_ = 41.5, p < 0.01, F_OEA_ = 15.2, p < 0.01; F_PEA_ = 12.7, p < 0.01) homogenates, with no differences among the three groups (Table 2). Results of post-hoc analyses are reported in Table 2.

**Figure 5:**
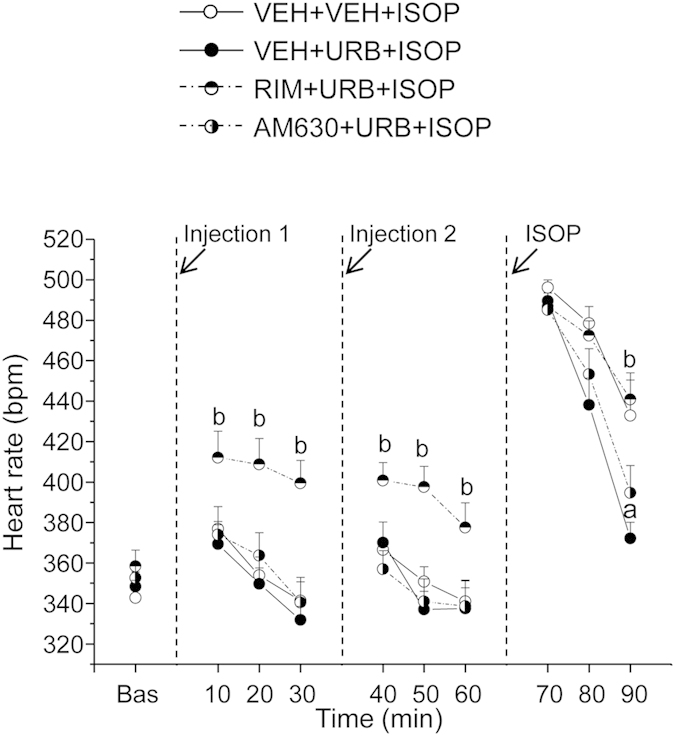
Time course of heart rate changes in HAB rats (n = 8) after the following sequences of intervention: (i) blockade of CB_1_ or CB_2_ receptors with rimonabant (RIM, 2 mg/kg) or AM630 (2 mg/kg), respectively, or vehicle (VEH) administration; (ii) inhibition of FAAH activity with URB694 (URB, 0.3 mg/kg) or VEH administration; (iii) β-adrenergic stimulation with isoproterenol (ISOP, 0.02 mg/kg). Data are reported as means ± SEM of a 30-min time period for baseline conditions (bas) and three 10-min time periods after each injection. VEH+VEH+ISOP group served as control for VEH+URB+ISOP group. VEH+URB+ISOP group served as control for RIM+URB+ISOP and AM630+URB+ISOP group. Significant differences (p < 0.05): ^a^vs. VEH+VEH+ISOP group; ^b^vs. VEH+URB+ISOP group.

**Figure 6:**
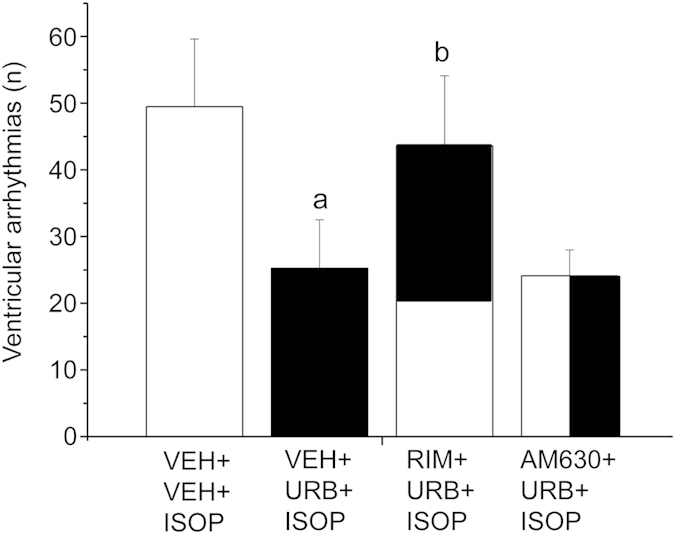
Incidence of ventricular tachyarrhythmias after β-adrenergic stimulation with isoproterenol (ISOP, 0.02 mg/kg) in HAB (n = 8). The injection of ISOP was preceded by inhibition of FAAH activity with URB694 (URB, 0.3 mg/kg) or vehicle (VEH) administration. URB was administered in the presence of CB_1_ or CB_2_ receptor blockade with rimonabant (RIM, 2 mg/kg) or AM630 (2 mg/kg), respectively, or after VEH injection. Data are reported as means ± SEM of a 30-min time period. VEH+VEH+ISOP group served as control for VEH+URB+ISOP group. VEH+URB+ISOP group served as control for RIM+URB+ISOP and AM630+URB+ISOP group. Significant differences (p < 0.05): ^a^vs. VEH+VEH+ISOP group; ^b^vs. VEH+URB+ISOP group.

**Experiment 3**. *Pharmacological challenge with isoproterenol*. HR and HRV changes in HAB rats during pharmacological challenge with ISOP are depicted in Fig. 5 and summarized in Table 3. In HAB rats, injection of RIM resulted in (i) higher HR values (t = 3.2, p < 0.01) (Fig. 5), (ii) a higher LF to HF ratio (t = 2.7, p < 0.05) (Table 3), and (iii) a larger incidence of ventricular arrhythmias (t = 3.3, p < 0.01) compared to VEH administration (Table 3). The tachycardic effect of RIM persisted after injection of URB (t = 3.3, p < 0.01) (Fig. 5). AM630 administration had no effects on HR and HRV parameters compared to VEH (Fig. 5 and Table 3). The peak HR reached during the first 10 min after ISOP injection was similar between the four groups (Fig. 5). However, during the last 10 min after ISOP administration HAB rats that had been pretreated with URB showed lower HR values (t = 2.9, p < 0.05) compared to VEH pretreatment condition (Fig. 5). This effect was prevented by administration of RIM (t = 2.9, p < 0.05), but not of AM630. In accordance with the results of Experiment 1, the incidence of ISOP-induced ventricular arrhythmias was significantly lower in HAB rats that had been pretreated with URB compared to VEH pretreatment condition (t = 3.0, p < 0.05) (Fig. 6). This effect was prevented by administration of RIM (t = 2.6, p < 0.05), but not of AM630 (Fig. 6).

## Discussion

The present study demonstrates that the heterogeneity in ventricular refractoriness is likely to represent the electrical substrate underlying the larger vulnerability to ventricular arrhythmias observed in rats with high levels of trait anxiety. Furthermore, we show that pharmacological inhibition of FAAH activity exerts anxiolytic-like effects and improves cardiac electrical stability in these animals.

The behavior of HAB/NAB/LAB rats on the elevated plus maze is consistent with extensive literature documenting clear differences in the level of anxiety among these rat lines^31^. Indeed, as expected, HAB rats spent significantly less time on the open/unprotected arms, which is interpreted as an index of high anxiety-related behavior^31^,^43^. Importantly, acute pre-treatment with URB694 prolonged the time spent by HAB rats on the open arms. This finding is consistent with previous preclinical studies demonstrating that genetic and pharmacological inhibition of FAAH activity exerts anxiolytic-like actions, likely via facilitation of brain AEA signaling at CB_1_ receptors^14^,^15^,^16^,^17^,^18^.

Acute administration of URB694 did not affect heart rate and indexes of heart rate variability in any of the three groups compared to vehicle administration, thus suggesting that pharmacological enhancement of AEA signaling does not influence central autonomic control of cardiac function. In addition, acute inhibition of FAAH activity did not influence the heart rate response to pharmacological stimulation of cardiac β-receptors with isoproterenol. We found that, similarly to a previous study^9^, HAB rats showed an increased vulnerability to ventricular tachyarrhythmias induced by isoproterenol. Most interestingly, arrhythmia vulnerability was significantly reduced in these animals by acute pre-treatment with URB694. Investigation of the potential underlying pro-arrhythmic electrical substrates clearly points to a larger dispersion of ventricular refractoriness in HAB rats. Heterogeneity of the effective refractory period duration among different ventricular epicardial sites reflects temporal differences in recovery of excitability and expresses electrical instability^42^. Importantly, heterogeneity of ventricular repolarization has also been reported in high-anxious human individuals^5^,^7^, thus highlighting the promising translational value of the HAB/LAB model for the study of the link between trait anxiety and cardiac electrical instability. Current available human data indicate that chronic anxiety may cause enduring changes in the cardiac sympathovagal balance towards sympathetic hyperactivity and/or parasympathetic hypoactivity^5^,^6^; this condition could promote left ventricular hypertrophy^44^. The abnormalities in potassium channels’ current induced by myocardial hypertrophy^45^, along with autonomic imbalance, might ultimately lead to exaggerated dispersion of ventricular repolarization in anxious subjects^5^,^7^. The cellular and sub-cellular bases of the electrical remodeling reported in HAB rats have not been investigated here and need further scrutiny. However, similarly to humans, HAB rats have recently been found to display decreased vagal modulation of sinus activity and morphological signs of cardiac hypertrophy^9^. Therefore, we hypothesize that the potassium current abnormalities associated with hypertrophic myocardial cells might represent a putative mechanism responsible for the prolonged refractory period and its exaggerated dispersion observed in this study in HAB rats. Heterogeneity in ventricular refractoriness is likely to play a role in both the initiation and the maintenance of ventricular arrhythmias by generating a substrate for functional re-entry^42^,^46^,^47^. Consequently, it is reasonable to deduce that in the presence of such pro-arrhythmic electrical substrate, a condition of potent β-adrenergic stimulation, such as it is isoproterenol administration, favors the occurrence of ventricular arrhythmias in HAB rats. Given the above discussed role of heterogeneity in ventricular refractoriness in creating a substrate for arrhythmias, the anti-arrhythmic effects of URB694 could likely be due to the ability of this drug to correct the exaggerated dispersion of ventricular refractoriness observed in HAB animals. Of note, inhibition of FAAH activity resulted also in a reduction in the spatial dispersion of ventricular excitability. Our hypothesis was that the anti-arrhythmic action of URB694 might be mediated by facilitation of AEA signaling at CB_1_ receptors within the heart tissue. Indeed, we found that URB694 administration, at the dose used in this study, almost completely abolished FAAH activity in heart homogenates. This consequently resulted in an increase in atrial and ventricular levels of AEA, and to a lesser extent of OEA and PEA. Moreover, the anti-arrhythmic effect of URB694 was prevented by pharmacological blockade of CB_1_, but not of the CB_2_, receptor. Interestingly, a previous study has demonstrated that AEA, in a concentration-dependent manner, decreases L-type calcium current in isolated rat ventricular myocytes via stimulation of CB_1_ receptors^48^. Based on the fact that (i) L-type calcium channels are the predominant mechanisms of calcium influx in cardiac cells^49^, and that (ii) blockade of these channels provides anti-arrhythmic actions^50^, the authors concluded that AEA may act as a cardiac protective factor through suppression of calcium influx^48^. In another study, the same research group found that AEA suppresses the outward potassium current (*I*_to_) in isolated rat ventricular myocytes through a non-CB_1_ and non-CB_2_ receptor-mediated pathway^51^. In rodents, *I*_to_ dominates the initial phase of repolarization^52^, and numerous studies have established that *I*_to_ reductions in rat myocardium cause prolongation of the action potential and refractory period^53^. Therefore, it may be that augmentation of AEA signaling within the heart tissue with URB694 provoked a prolongation of the effective refractory period by suppressing *I*_to_. Importantly, inhibition of *I*_to_ is considered an effective strategy for preventing/suppressing re-entrant arrhythmias^54^. The results of the cited studies may offer a mechanistic interpretation of the anti-arrhythmic effects of URB694 in rats exhibiting heightened anxiety-like behavior.

In addition, administration of the CB_1_ antagonist rimonabant provoked a larger tachycardic response and a larger shift of the sympathovagal balance towards sympathetic prevalence (LF to HF ratio) in HAB rats compared to vehicle administration. This result is in line with previous observations that ECB-mediated activation of pre-synaptic CB_1_ receptors is critical for modulating stress-induced sympathetic nervous system activation, via down-regulation of excitatory (e.g. glutamatergic) neurotransmission^55^,^56^. Compared to vehicle, the CB_2_ antagonist AM630 had no effect on heart rate, nor did it influence the faster heart rate recovery from isoproterenol challenge observed after URB694 administration. This rules out a role of CB_2_ receptors on URB694 activity. On the other hand, the intrinsic effect of rimonabant on heart rate precluded definitive assessment of the role of CB_1_ receptors in modulating isoproterenol-induced tachycardia. Interestingly, blockade of CB_1_ receptors with rimonabant also caused a larger incidence of ventricular tachyarrhythmias in HAB rats compared to vehicle administration. Even though the incidence of arrhythmias reported here after CB_1_ receptor blockade was rather moderate, this finding suggests that ECB signaling within the heart tissue might play an important role in the maintenance of cardiac homeostasis under stressful challenges.

In conclusion, the present study extends previous research on the integration of anxiety and cardiac dysfunction by describing the electrical pro-arrhythmic remodeling in rats selectively bred for high-trait anxiety. Heterogeneity in ventricular refractoriness is regarded here as the major determinant of the increased vulnerability to ventricular arrhythmias that characterizes HAB rats. Furthermore, the results of this study suggest that inhibition of FAAH with URB694 exerts anxiolytic-like and cardioprotective effects, the latter likely via a CB_1_ receptor-mediated mechanism. Our findings have to be regarded with several major limitations in mind: (i) we did not assess whether higher or lower doses of URB694 can affect cardiac function in a dose-dependent fashion, (ii) we did not clarify whether the cardioprotective effects of URB694 are mediated by facilitation of CB_1_ receptor-mediated AEA signaling at the peripheral and/or central level (for instance, this could be achieved by inhibiting FAAH with an URB694-like molecule that does not cross the blood brain barrier), and (iii) we did not thoroughly investigated the effects of CB_1_ and CB_2_ receptor blockade with rimonabant and AM630, respectively, on cardiac function. Nevertheless, we believe that these findings represent an important first step towards an understanding of the potential anxiolytic-like and cardioprotective properties of FAAH inhibitors in preclinical models of anxiety.

## Additional Information

**How to cite this article**: Carnevali, L. *et al.* Cardioprotective effects of fatty acid amide hydrolase inhibitor URB694, in a rodent model of trait anxiety. *Sci. Rep.* **5**, 18218; doi: 10.1038/srep18218 (2015).

## Figures and Tables

**Table 1:** Heart rate, heart rate variability parameters and incidence of ventricular arrhythmias in HAB, NAB and LAB rats (n = 9 per group) in baseline conditions and in response to (i) vehicle (VEH) + isoproterenol (ISOP, 0.02 mg/kg) injections (left columns), and (ii) URB694 (URB, 0.3 mg/kg) + ISOP injections (right columns).

Heart rate (bpm)	HAB	NAB	LAB	Heart rate (bpm)	HAB	NAB	LAB
Basal (30 min)	352 ± 11	364 ± 6	364 ± 13	Basal (30 min)	355 ± 7	383 ± 12	363 ± 17
VEH (30 min)	359 ± 9^ab^	401 ± 9	403 ± 13	URB (30 min)	348 ± 6^ab^	404 ± 11	398 ± 11
ISOP (30 min)	476 ± 9	477 ± 8	494 ± 9	ISOP (30 min)	467 ± 11	484 ± 7	477 ± 19
**RMSSD (ms)**	**HAB**	**NAB**	**LAB**	**RMSSD (ms)**	**HAB**	**NAB**	**LAB**
Basal (30 min)	4.0 ± 0.4	4.0 ± 0.3	4.5 ± 0.6	Basal (30 min)	4.0 ± 0.4	3.4 ± 0.3	4.2 ± 0.4
VEH (30 min)	3.9 ± 0.3	3.9 ± 0.3	4.1 ± 0.4	URB (30 min)	3.8 ± 0.4	3.2 ± 0.4	3.9 ± 0.4
ISOP (30 min)	1.8 ± 0.2	2.6 ± 0.2	2.3 ± 0.2	ISOP (30 min)	2.0 ± 0.2	2.3 ± 0.5	2.0 ± 0.4
**HF (ms**^**2**^)	**HAB**	**NAB**	**LAB**	**HF (ms**^**2**^)	**HAB**	**NAB**	**LAB**
Basal (30 min)	5.9 ± 1.0	5.3 ± 0.8	7.7 ± 2.4	Basal (30 min)	5.7 ± 1.1	4.3 ± 0.8	6.5 ± 1.4
VEH (30 min)	5.2 ± 0.7	5.3 ± 0.7	6.8 ± 0.9	URB (30 min)	5.5 ± 1.0	3.8 ± 1.1	5.8 ± 1.5
ISOP (30 min)	1.5 ± 0.3	2.8 ± 0.6	2.3 ± 0.3	ISOP (30 min)	2.0 ± 0.5	2.7 ± 1.2	2.2 ± 0.7
**LF/HF**	**HAB**	**NAB**	**LAB**	**LF/HF**	**HAB**	**NAB**	**LAB**
Basal (30 min)	0.8 ± 0.1	0.6 ± 0.1	0.6 ± 0.1	Basal (30 min)	0.8 ± 0.1	0.7 ± 0.1	0.8 ± 0.1
VEH (30 min)	0.9 ± 0.1	0.7 ± 0.1	0.8 ± 0.1	URB (30 min)	0.8 ± 0.1	0.9 ± 0.1	1.0 ± 0.1
ISOP (30 min)	0.7 ± 0.1	0.6 ± 0.1	0.7 ± 0.1	ISOP (30 min)	0.7 ± 0.1	0.6 ± 0.1	0.8 ± 0.1
**Arrhythmias (n)**	**HAB**	**NAB**	**LAB**	**Arrhythmias (n)**	**HAB**	**NAB**	**LAB**
Basal (30 min)	0.2 ± 0.2	0.1 ± 0.1	0.0 ± 0.0	Basal (30 min)	0.0 ± 0.0	0.0 ± 0.0	0.0 ± 0.0
VEH (30 min)	2.3 ± 2.2	0.4 ± 0.2	0.1 ± 0.1	URB (30 min)	0.3 ± 0.2	0.4 ± 0.3	0.0 ± 0.0
ISOP (30 min)	41.7 ± 12.3^ab^	11.8 ± 2.5	12.3 ± 2.6	ISOP (30 min)	14.1 ± 4.2*	8.4 ± 2.2	7.3 ± 3.7

Data are reported as means ± SEM of the indicated time periods. Significant differences (p < 0.05): ^a^vs. NAB value, ^b^vs. LAB value, *vs. respective control value.

**Table 2:** FAAH activity and anandamide (AEA), oleoylethanolamide (OEA) and palmitoylethanolamide (PEA) levels in atrial and ventricular homogenates of HAB, NAB and LAB rats (n = 6–8 per group).

FAAH atrial activity (cpm)	VEH	URB	FAAH ventricular activity (cpm)	VEH	URB
HAB	197.0 ± 84.1^ab^	29.8 ± 22.4*	HAB	196.0 ± 17.0^a^	30.0 ± 13.6*
NAB	851.3 ± 159.1	36.3 ± 13.5*	NAB	478.9 ± 165.9	33.1 ± 21.0*
LAB	520.3 ± 90.5^a^	30.3 ± 34.3*	LAB	345.4 ± 112.0	34.3 ± 12.3*
**AEA atrial levels (pmol/g)**	**VEH**	**URB**	**AEA ventricular levels (pmol/g)**	**VEH**	**URB**
HAB	14.6 ± 1.1	23.3 ± 2.7*	HAB	29.0 ± 2.1	45.6 ± 4.7*
NAB	15.6 ± 1.6	23.9 ± 2.9*	NAB	24.7 ± 1.1	48.7 ± 2.5*
LAB	18.6 ± 1.2	24.6 ± 1.7*	LAB	33.1 ± 1.8^a^	46.7 ± 7.9
**OEA atrial levels (pmol/g)**	**VEH**	**URB**	**OEA ventricular levels (pmol/g)**	**VEH**	**URB**
HAB	111.6 ± 6.7	152.9 ± 31.8	HAB	102.8 ± 8.9^a^	128.2 ± 19.8
NAB	101.5 ± 7.2	154.7 ± 27.2	NAB	75.5 ± 3.6	117.4 ± 15.5*
LAB	101.7 ± 8.2	138.7 ± 19.2	LAB	97.9 ± 10.3^a^	110.8 ± 24.1
**PEA atrial levels (pmol/g)**	**VEH**	**URB**	**PEA ventricular levels (pmol/g)**	**VEH**	**URB**
HAB	111.3 ± 7.9	144.8 ± 32.3	HAB	124.3 ± 8.3	149.2 ± 24.4
NAB	105.2 ± 8.7	149.5 ± 16.2*	NAB	125.9 ± 9.0	146.5 ± 15.9
LAB	111.7 ± 8.9	151.5 ± 9.6*	LAB	112.4 ± 7.8	122.5 ± 27.9

FAAH activity and AEA, OEA and PEA levels were assessed 60 min after administration of vehicle (VEH) or URB694 (URB, 0.3 mg/kg). Data are reported as means ± SEM. Significant differences (p < 0.05): ^a^vs. NAB value, ^b^vs. LAB value,*vs. respective control (VEH) value.

**Table 3:** Heart rate, heart rate variability parameters and incidence of ventricular arrhythmias in HAB rats (n = 8) in baseline conditions and in response to the following sequences of injections: (i) vehicle (VEH) + VEH + isoproterenol (ISOP, 0.02 mg/kg), (ii) VEH + URB694 (URB, 0.3 mg/kg) + ISOP, (iii) rimonabant (RIM, 2 mg/kg) + URB + ISOP, and (iv) AM630 (2 mg/kg) + URB + ISOP.

Heart rate (bpm)	VEH + VEH + ISOP	VEH + URB + ISOP	RIM + URB + ISOP	AM630 + URB + ISOP
Basal (30 min)	343 ± 9	349 ± 7	358 ± 8	353 ± 7
Injection 1 (30 min)	356 ± 10	350 ± 5	404 ± 11^b^	359 ± 8
Injection 2 (30 min)	353 ± 6	348 ± 10	392 ± 10^b^	346 ± 9
ISOP (30 min)	462 ± 9	417 ± 11^a^	462 ± 7^b^	438 ± 8
**RMSSD (ms)**	**VEH** + **VEH** + **ISOP**	**VEH** + **URB** + **ISOP**	**RIM** + **URB** + **ISOP**	**AM630** + **URB** + **ISOP**
Basal (30 min)	2.8 ± 0.2	2.9 ± 0.3	2.8 ± 0.3	3.1 ± 0.3
Injection 1 (30 min)	2.7 ± 0.2	3.0 ± 0.3	2.8 ± 0.3	2.8 ± 0.3
Injection 2 (30 min)	2.7 ± 0.2	3.0 ± 0.4	2.9 ± 0.3	2.9 ± 0.3
ISOP (30 min)	1.6 ± 0.2	2.5 ± 0.2^a^	1.6 ± 0.2^b^	1.6 ± 0.2
**HF (ms**^**2**^)	**VEH** + **VEH** + **ISOP**	**VEH** + **URB** + **ISOP**	**RIM** + **URB** + **ISOP**	**AM630** + **URB** + **ISOP**
Basal (30 min)	2.8 ± 0.4	2.9 ± 0.7	2.8 ± 0.6	3.5 ± 0.7
Injection 1 (30 min)	2.5 ± 0.4	3.2 ± 0.5	2.5 ± 0.5	3.1 ± 0.6
Injection 2 (30 min)	2.3 ± 0.3	3.3 ± 0.8	3.0 ± 0.6	2.8 ± 0.5
ISOP (30 min)	1.2 ± 0.2	2.5 ± 0.4^a^	1.2 ± 0.3^b^	1.7 ± 0.2
**LF/HF**	**VEH** + **VEH** + **ISOP**	**VEH** + **URB** + **ISOP**	**RIM** + **URB** + **ISOP**	**AM630** + **URB** + **ISOP**
Basal (30 min)	0.7 ± 0.1	0.8 ± 0.1	0.8 ± 0.1	0.8 ± 0.1
Injection 1 (30 min)	1.0 ± 0.1	0.8 ± 0.1	1.2 ± 0.1^b^	1.0 ± 0.1
Injection 2 (30 min)	1.0 ± 0.1	0.9 ± 0.1	1.1 ± 0.1	0.9 ± 0.1
ISOP (30 min)	0.7 ± 0.1	0.8 ± 0.1	0.6 ± 0.1	0.8 ± 0.1
**Arrhythmias (n)**	**VEH** + **VEH** + **ISOP**	**VEH** + **URB** + **ISOP**	**RIM** + **URB** + **ISOP**	**AM630** + **URB** + **ISOP**
Basal (30 min)	0.3 ± 0.2	0.3 ± 0.2	0.4 ± 0.2	0.4 ± 0.3
Injection 1 (30 min)	0.4 ± 0.2	0.3 ± 0.3	2.9 ± 0.8^b^	0.6 ± 0.3
Injection 2 (30 min)	0.3 ± 0.3	0.3 ± 0.2	2.4 ± 1.2	0.3 ± 0.3
ISOP (30 min)	49.5 ± 10.2	25.3 ± 7.3^a^	43.6 ± 10.5^b^	24.1 ± 3.9

Data are reported as means ± SEM of the indicated time periods. VEH + VEH + ISOP group served as control for VEH + URB + ISOP group. VEH + URB + ISOP group served as control for RIM + URB + ISOP and AM630 + URB + ISOP group. Significant differences (p < 0.05): ^a^vs. VEH + VEH + ISOP group; ^b^vs. VEH + URB + ISOP group.
